# Medication adherence with denosumab in patients with bone metastases from solid tumors treated in routine clinical settings: a retrospective study

**DOI:** 10.1007/s00520-022-07333-7

**Published:** 2022-09-06

**Authors:** Ingo J. Diel, Richard Greil, Jan Janssen, Christian W. Kluike, Bagmeet Behera, Ali Abbasi, Anouchka Seesaghur, Michael Kellner, Christine Jaeger, Katja Bjorklof, Antoaneta Tomova, Ferdinand Haslbauer

**Affiliations:** 1 Praxisklinik Am Rosengarten, Augustaanlage 7–11, 68165 Mannheim, Germany; 2 Paracelsus Medizinische Privatuniversität, Salzburg, Austria; 3 Salzburg Cancer Research Institute-Center for Clinical Cancer and Immunology Trials and Cancer Cluster, Salzburg, Austria; 4 Medizinische Studiengesellschaft Nord-West GmbH, Westerstede, Germany; 5 Praxis für Urologie Kluike und Weiler, Lüneburg, Germany; 6 Amgen Research Munich GmbH, Munich, Germany; 7 Center for Observational Research, Amgen, Uxbridge, UK; 8 Amgen GmbH, Munich, Germany; 9 Amgen GmbH, Vienna, Austria; 10 Amgen Europe GmbH, Rotkreuz, Switzerland; 11 Complex Oncology Center Plovdiv EOOD, Plovdiv, Bulgaria; 12 Salzkammergut Klinikum Vöcklabruck, Vöcklabruck, Austria

**Keywords:** Medication adherence, Initiation, Implementation, Persistence, Denosumab, Real-world study

## Abstract

**Purpose:**

To describe (non)adherence with denosumab among patients with solid tumors and bone metastases.

**Methods:**

This retrospective, observational study pooled data from two completed prospective, multicenter cohort studies (X-TREME; Study 240) in adult patients with bone metastases from primary breast, prostate, lung, kidney, or other solid cancer types and administered denosumab 120 mg in routine clinical practice in Germany and Central and Eastern Europe. The studies were conducted between May 2012 and May 2017; pooled analysis was completed in August 2021. Medication adherence was described according to a three-component consensus taxonomy: initiation (first-ever administration ≤ 90 days from bone metastasis diagnosis), implementation (actual vs prescribed dosing; optimal implementation = regular/consistent dosing), and persistence (≤ 60-day gap between administrations at 3, 6, 9, and 12 months). Descriptive analyses were conducted for each cancer type.

**Results:**

The analysis included 1748 patients with solid tumors and bone metastases. Adherence with denosumab was generally high across the initiation, implementation, and persistence phases. Most patients experienced timely initiation (from 64.4% [kidney cancer] to 81.2% [breast cancer]) and optimal implementation (from 62.4% [lung cancer] to 72.5% [breast cancer]). The proportion of patients who were persistent with treatment at 6 months ranged from 41.4% (lung cancer) to 77.8% (prostate cancer).

**Conclusions:**

This study revealed variations by cancer type in the initiation, implementation, and persistence of denosumab in patients with solid tumors and bone metastases in routine clinical practice. Further cancer-specific studies are warranted to examine the determinants of (non)adherence with denosumab, and potential ways to improve medication adherence.

**Supplementary Information:**

The online version contains supplementary material available at 10.1007/s00520-022-07333-7.

## Introduction

Bone metastases (BMs) are common in patients with solid tumors [1, 2]. Early treatment to prevent skeletal-related events (SREs), a debilitating complication of BMs, is crucial [3]. European Society for Medical Oncology (ESMO) guidelines recommend that bone-targeted agents (BTAs) are initiated as soon as BMs are diagnosed and continued indefinitely throughout the course of the disease [2]. Denosumab is a fully human monoclonal antibody that inhibits the receptor activator of nuclear factor κB ligand (RANKL) on bone cells [4]. It is indicated for the prevention of SREs (pathological fracture, radiation to bone, spinal cord compression, or surgery to bone) in adults with advanced malignancies involving bone [5]. Denosumab is given subcutaneously with a recommended schedule of every 4 weeks.

Adhering to medication is the process by which patients take their medication per label and is essential for optimal therapeutic benefit [6–8]. Failure to comply with the recommended regimen or failure to persist with therapy is an important determinant of therapeutic non-response. To ensure optimal adherence to medication, it is important to understand the magnitude of nonadherence in a population. Persistence with denosumab has been described in previous studies [4, 9–12]; however, inconsistency in definitions and varying analytic approaches hamper data interpretation [6, 13]. Optimal assessment of medication adherence requires robust operational definitions and methods. A three-component consensus taxonomy for describing phases of medication adherence (initiation, implementation, and persistence) has been recommended by the European Society for Patient Adherence, COMpliance, and Persistence (ESPACOMP) [6]. *Initiation* relates to the first-ever administration, *implementation* is based on actual dosing versus prescribed/on-label dosing, and *persistence* is the period between initiation and the last dose [8]. Conversely, medication nonadherence is described as late/non-initiation, suboptimal implementation, or non-persistence (early discontinuation) [6, 8].

The objective of this retrospective, observational analysis using pooled data from two completed prospective, multicenter cohort studies [4, 9, 10, 14] was to describe (non)adherence with denosumab (initiation, implementation, and persistence) in patients diagnosed with solid tumors and BMs in routine clinical practice.

## Methods

### Study design

This was a retrospective, observational study using pooled data from two completed prospective, observational, multicenter studies in patients with solid tumors and BMs treated with denosumab [4, 9, 10, 14]. X-TREME (Study 20101312) was conducted in Germany [9, 14], whereas Study 240 (20110240) was conducted in Austria, Bulgaria, Czech Republic, Hungary, and Slovakia [4, 10]. Both studies evaluated the persistence of treatment with denosumab in routine clinical practice, with patients receiving treatment as per routine clinical practice in the respective countries [4, 9, 10, 14].

The overall observation period for this analysis was from May 7, 2012, to May 26, 2017 (X-TREME: May 7, 2012, to January 12, 2017; Study 240: October 4, 2012, to May 26, 2017). The study designs for the original studies are summarized in Supplementary Fig. 1 [4, 9]. Further details have been published elsewhere [4, 9, 10, 14].

Written informed consent was provided by all patients before recruitment in the original studies or any data collection. The original study protocols and informed consent forms were approved by an investigational review board, independent ethics committee, or relevant country-specific authorities and conducted in accordance with the principles of the Declaration of Helsinki.

### Eligibility criteria

In the present analysis, all patients from X-TREME and Study 240 were included, except for those whose first-ever denosumab administration could not be ascertained. The aggregated study database contained the electronic case report forms completed by physicians and questionnaires completed by patients. The original studies comprised adult patients (aged ≥ 18 years) who had been diagnosed with a solid tumor and BMs, treated with denosumab (per routine standard practice and per label-recommended dose of 120 mg administered as a single subcutaneous injection once every 4 weeks), and had an Eastern Cooperative Oncology Group performance status score of 0–2 at study enrollment. Prior treatment with bisphosphonates or other antiresorptive therapy for SRE prevention was permitted so long as the duration did not exceed 6 months.

### Measurements and definitions

**Fig. 1:**
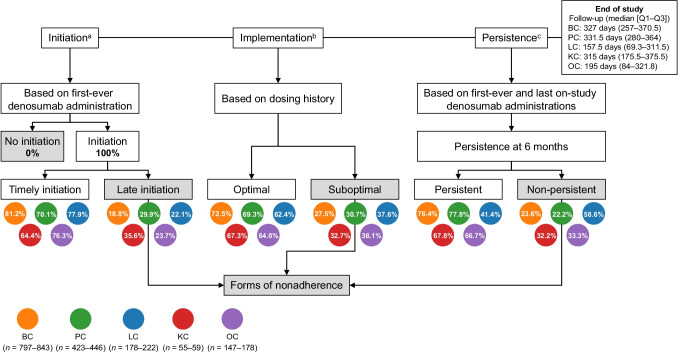
Forms of adherence and nonadherence with denosumab. Percentages within colored circles denote the proportion of patients by cancer type. Please refer to Table 2 and Supplementary Fig. 2 for additional data and full adherence definitions. ^a^Time to initiation was defined as the time from diagnosis of bone metastasis to first-ever denosumab administration. ^b^Each denosumab administration for patients with ≥ 3 doses of denosumab. Patients classified as optimal or suboptimal according to the extent to which gaps between administrations correspond to the recommended administration gap of 28 days, in terms of both regularity and consistency. ^c^No gap of > 60 days between consecutive denosumab administrations from initiation. *BC* breast cancer, *KC* kidney cancer, *LC* lung cancer, *OC* other types of cancer, *PC* prostate cancer

The three phases of medication adherence—initiation, implementation, and persistence—were measured (Fig. 1; details in Supplementary Table 1 [6]). Forms of nonadherence were late initiation, suboptimal implementation, or non-persistence.

The timing of the first-ever denosumab administration relative to BM diagnosis was based on ESMO recommendations [2], and an arbitrary 90-day cutoff after BM diagnosis was based on use in analyses in previous studies and on the guidance of independent clinicians [15, 16].

Optimal implementation of the denosumab dosing regimen was defined as regular and consistent dosing, and suboptimal implementation was defined as irregular or inconsistent dosing. Regular dosing occurred when the mean dose gap was ≤ 28 days, and consistent dosing when there was no large (≤ 10%) deviation from the mean dose gap. Irregular dosing occurred when the mean dose gap was > 28 days, and inconsistent dosing when there were large deviations from the mean dose gap.

Persistence was defined as the time from the first-ever denosumab administration to the discontinuation date (the last record of denosumab administration before a 60-day gap between consecutive administrations), lost to follow-up, switch to another therapy, or end of the study period. The proportion of patients who were persistent with denosumab after initiation (i.e., no gap > 60 days between administrations) at 3, 6, 9, and 12 months was calculated. Patients were defined as non-persistent at 3, 6, 9, and 12 months if they had a > 60-day gap between consecutive administrations, refused further denosumab treatment, discontinued treatment because of a reportable adverse drug reaction, or was lost to follow-up (for any reason including death) or if the physician stopped treatment. Although ESMO guidelines recommend usually continuing BTAs indefinitely, the observation (pre-defined end-of-study) period was limited to 52 weeks for X-TREME and 48 weeks for Study 240. While most patients were not followed up beyond these time frames, some patients continued treatment beyond the end-of-study timepoint and were followed until they stopped treatment, were censored, or died. This allowed persistence to be reported at 12 months. Due to the large number of patients who were lost to follow-up and the high risk of death for cancer patients with metastasis, the true long-term persistence of patients on denosumab cannot be assessed.

Patient characteristics, comorbidities, and medications, as well as patient-reported outcomes (PROs), were assessed. PROs were measured using the EuroQoL 5-Dimension 5-Level questionnaire that comprised dimensions including pain, mobility, self-care, and usual activities. Information on pain medications was collected before enrollment and at 3 months after the first denosumab dose.

### Statistical analysis

De-identified, patient-level data from the two studies were combined into a single dataset. Given the descriptive nature of most analyses, no statistical comparisons or multivariate modeling was performed. Descriptive analyses summarized patient characteristics, including frequencies (%) for categorical variables, and mean (standard deviation) and median (Q1–Q3) for continuous variables. Phases of adherence/nonadherence were analyzed as binary outcomes. Each adherence category was described by cancer type.

Medication persistence was an estimate of the proportion of patients with continuous denosumab administration (i.e., no gap > 60 days between administrations) after denosumab initiation over the study period. The Aalen–Johansen estimator was used for deriving the cumulative risk (*F[t, j]*) of non-persistence without censoring the competing risk events:

$$\widehat{F}\left(t,j\right)=\sum_{k\le t}\frac{{d}_{kj}}{{n}_{k}}\widehat{S}(k-1)$$

where *d*_*kj*_ is the number of events of type *j* occurring at time *k*, *n*_*k*_ is the number of individuals at risk of the event at time *k*, *d*_*kj*_*/n*_*k*_ is the cause-specific hazard for the event of interest at time *k*, and *Ŝ(k − 1)* is an estimate of overall survival function at the previous time-point [17]. The cumulative risk of both non-persistence and death was quantified for each cancer type. Python programming language (version 3.7.6) and the standard Python numeric packages NumPy and Pandas were used for all analyses. The “AalenJohansenFitter” from the Python Lifelines package was used for calculating cumulative risks.

## Results

### Patient demographics and clinical characteristics

The analysis included a total of 1748 adult patients with solid tumors and BMs, including 843 patients with breast cancer, 446 with prostate cancer, 222 with lung cancer, 59 with kidney cancer, and 178 with other types of cancer (Table 1). The mean age was between 62.5 and 72.7 years across cancer types, and the median duration of follow-up from the first-ever dose of denosumab was between 157.5 and 331.5 days. Over 70% of patients had a history of anticancer therapy, and between 4.1% and 10.2% of patients across cancer types had prior antiresorptive therapy. Data on bone pain or history of SRE before study enrollment was unknown/not collected for many patients. The proportion of patients with more than one BM at baseline was 71.0% (breast cancer), 78.5% (prostate cancer), 59.9% (lung cancer), 52.5% (kidney cancer), and 55.4% (other types of cancer). Common comorbidities were diabetes (6.8–14.7%), chronic kidney disease (3.9–30.5%), and cardiovascular disease (up to 15%) (Supplementary Table 2).

**Table 1:** Baseline demographics and clinical characteristics of patients by type of cancer

Characteristic	Breast cancer	Prostate cancer	Lung cancer	Kidney cancer	Other types of cancer
Age (years)					
Patients, *n*	843	446	222	59	178
Mean (SD)	62.5 (11.8)	72.7 (8.1)	64.4 (9.5)	66.9 (9.5)	65.4 (10.4)
Sex					
Patients, *n*	843	446	222	59	178
Female	843 (100.0)	0 (0)	79 (35.6)	22 (37.3)	78 (43.8)
Country					
Patients, *n*	843	446	222	59	178
Germany	506 (60.0)	296 (66.4)	159 (71.6)	50 (84.7)	116 (65.2)
Austria	197 (23.4)	28 (6.3)	36 (16.2)	2 (3.4)	45 (25.3)
Eastern Europe^a^	140 (16.6)	122 (27.4)	27 (12.2)	7 (11.9)	17 (9.6)
Prior medications					
Patients, *n*	842	441	222	59	177
Anticancer therapy	597 (70.9)	320 (72.6)^b^	172 (77.5)^c^	47 (79.7)	128 (72.3)^c^
Chemotherapy	312 (37.1)^d^	78 (17.7)^e^	148 (66.7)^f^	37 (62.7)^g^	104 (58.8)^h^
Antiresorptive therapy^i^	63 (7.5)	38 (8.6)^b^	9 (4.1)^c^	6 (10.2)	10 (5.6)^c^
Renal impairment at enrollment					
Patients, *n*	842	441	222	59	177
Yes	12 (1.4)	22 (5.0)	5 (2.3)	3 (5.1)	3 (1.7)
No	322 (38.2)	122 (27.7)	57 (25.7)	6 (10.2)	57 (32.2)
Not available	508 (60.3)	297 (67.3)	160 (72.1)	50 (84.7)	117 (66.1)
Hypocalcemia/hypercalcemia at enrollment					
Patients, *n*	842	441	222	59	177
Yes	18 (2.1)	6 (1.4)	4 (1.8)	2 (3.4)	6 (3.4)
No	316 (37.5)	137 (31.1)	58 (26.1)	7 (11.9)	54 (30.5)
Not available	508 (60.3)	298 (67.6)	160 (72.1)	50 (84.7)	117 (66.1)
Prior hypercalcemia					
Patients, *n*	842	441	222	59	177
Yes	22 (2.6)	9 (2.0)	5 (2.3)	4 (6.8)	7 (4.0)
No	373 (44.3)	151 (34.2)	77 (34.7)	19 (32.2)	63 (35.6)
Not available	447 (53.1)	281 (63.7)	140 (63.1)	36 (61.0)	107 (60.5)
Number of bone metastases					
Patients, *n*	842	441	222	59	177
1	188 (22.3)	77 (17.5)	78 (35.1)	25 (42.4)	67 (37.9)
2–4	313 (37.2)	157 (35.6)	83 (37.4)	20 (33.9)	62 (35.0)
> 4	283 (33.6)	189 (42.9)	50 (22.5)	11 (18.6)	36 (20.3)
Not available^j^	58 (6.9)	18 (4.1)	11 (5.0)	3 (5.1)	12 (6.8)
Bone pain					
Patients, *n*	842	441	222	59	177
Yes	40 (4.8)	13 (2.9)	14 (6.3)	11 (18.6)	8 (4.5)
No	63 (7.5)	10 (2.3)	13 (5.9)	5 (8.5)	12 (6.8)
Not available	739 (87.8)	418 (94.8)	195 (87.8)	43 (72.9)	157 (88.7)
History of SRE prior to enrollment					
Patients, *n*	842	441	222	59	177
≥ 1 record of specific SRE	91 (10.8)	20 (4.5)	25 (11.3)	13 (22.0)	18 (10.2)
No SRE recorded	299 (35.5)	141 (32.0)	57 (25.7)	9 (15.3)	52 (29.4)
SRE not available	452 (53.7)	280 (63.5)	140 (63.1)	37 (62.7)	107 (60.5)

*SRE* skeletal-related event. Data shown as *n* (%) unless indicated otherwise. ^a^Data for Bulgaria, Czech Republic, Hungary, and Slovakia were pooled because statistics for some tumor types were too low to be presented for the individual countries; data not available for ^b^2 patients, ^c^1 patient, ^d^134 patients, ^e^45 patients, ^f^23 patients, ^g^5 patients, and ^h^22 patients; ^i^in Study 240, prior antiresorptive therapy consisted of bisphosphonates, including zoledronic acid, ibandronate, pamidronate, and unspecified others [4]; in X-TREME, prior antiresorptive therapy included bisphosphonates; ^j^unknown or missing

### Medication adherence and nonadherence with denosumab

The median (Q1–Q3) time from BM diagnosis to denosumab initiation ranged from 30 (14–87) days (other types of cancer) to 51 (20–124) days (kidney cancer) (Table 2). Most patients with solid tumors and BMs experienced timely initiation (up to 81.2%) and optimal implementation (up to 72.5%) of denosumab. Persistence with denosumab ranged from 70.3% (lung cancer) to 86.8% (breast cancer) at 3 months, and from 13.5% (lung cancer) to 36.6% (prostate cancer) at 12 months.

**Table 2:** Adherence with denosumab by type of cancer

Phase of medication adherence/nonadherence	Breast cancer	Prostate cancer	Lung cancer	Kidney cancer	Other types of cancer
Initiation					
Patients, *n*	842	441	222	59	177
Time from bone metastasis diagnosis to denosumab initiation, median (Q1–Q3) days	32 (15–70)	43 (16–146)	32 (15–79)	51 (20–124)	30 (14–87)
Adherence: timely initiation	684 (81.2)	309 (70.1)	173 (77.9)	38 (64.4)	135 (76.3)
Nonadherence: late initiation	158 (18.8)	132 (29.9)	49 (22.1)	21 (35.6)	42 (23.7)
Implementation					
Patients, *n*	797	423	178	55	147
Adherence: optimal implementation	578 (72.5)	293 (69.3)	111 (62.4)	37 (67.3)	94 (64.0)
Nonadherence: suboptimal implementation	219 (27.5)	130 (30.7)	67 (37.6)	18 (32.7)	53 (36.1)
Persistence					
Patients, *n*	843	446	222	59	178
Adherence: persistent					
3 months	732 (86.8)	386 (86.6)	156 (70.3)	50 (84.8)	128 (71.9)
6 months	644 (76.4)	347 (77.8)	92 (41.4)	40 (67.8)	90 (50.6)
9 months	558 (66.2)	309 (69.3)	71 (32.0)	30 (50.9)	62 (34.8)
12 months	283 (33.6)	163 (36.6)	30 (13.5)	18 (30.5)	26 (14.6)
Nonadherence: non-persistent					
3 months	111 (13.2)	60 (13.5)	66 (29.7)	9 (15.3)	50 (28.1)
6 months	199 (23.6)	99 (22.2)	130 (58.6)	19 (32.2)	88 (49.4)
9 months	285 (33.8)	137 (30.7)	151 (68.0)	29 (49.2)	116 (65.2)
12 months	560 (66.4)	283 (63.5)	192 (86.5)	41 (69.5)	152 (85.4)

Data shown as *n* (%) unless indicated otherwise

Initiation of denosumab was delayed in 18.8% to 35.6% of patients with solid tumors and BMs across different cancer types, and implementation was suboptimal (irregular or inconsistent dosing) in 27.5% (breast cancer) to 37.6% (lung cancer; Table 2). Non-persistence with denosumab ranged from 13.2% (breast cancer) to 29.7% (lung cancer) at 3 months, and from 63.5% (prostate cancer) to 86.5% (lung cancer) at 12 months.

The incidence of death in the study cohort was high enough to compete with non-persistence events since death precludes non-persistence from occurring. This necessitates the risk of death to be described along with the risk of non-persistence. In patients with breast, prostate, or kidney cancer and associated BMs who had received denosumab, the risk of death during follow-up, as quantified by cumulative incidence (Supplementary Fig. 2), was similar to the risk of non-persistence, that is, the 95% confidence interval around the respective cumulative risks has overlap. In patients with lung cancer and BM, the incidence of death during follow-up was substantially higher than non-persistence. Across all cancer types, the number of patients who died within 600 days after denosumab initiation (*n* = 303) was 22% more than the number who were non-persistent (*n* = 248). The cumulative risk of non-persistence across cancer types and the cumulative risk of non-persistence and death in patients with breast cancer are shown in Supplementary Fig. 3 and Supplementary Fig. 4, respectively. The cumulative incidence of non-persistence alone remained very low during the study (between 0.15 and 0.20 for patients with prostate, lung, and other types of cancer, and < 0.35 for patients with breast and kidney cancer).

### Patient-reported quality of life

**Fig. 2:**
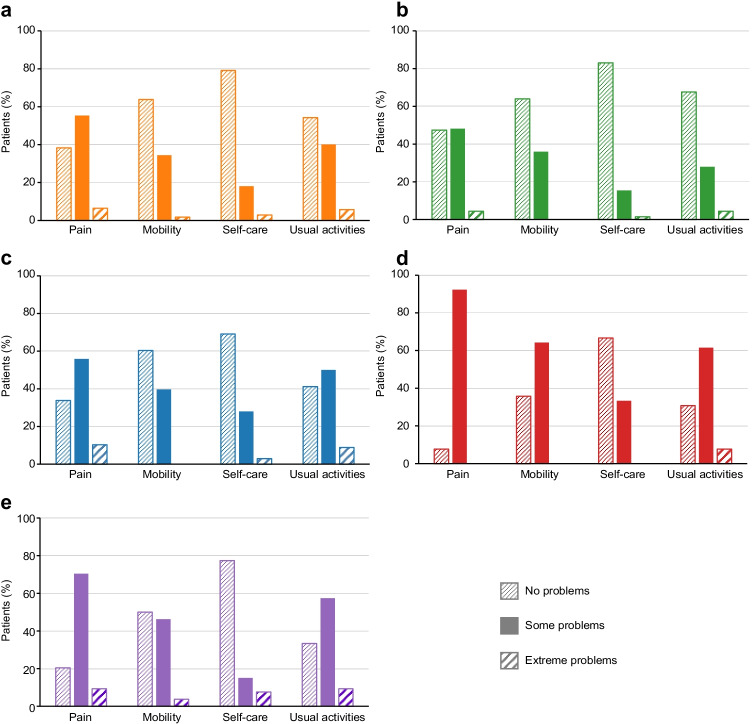
Quality of life assessed at month 3 of denosumab initiation via the EQ-5D-5L questionnaire domains in patients with **a** breast, **b** prostate, **c** lung, **d** kidney, and **e** other cancer types. Patients may have received radiotherapy for palliative pain. **a** Twenty-two point seven percent and 30.3% of patients had a history of pain medication (analgesics) prior to enrollment and at 3 months after the first dose of denosumab, respectively; 81.2% initiated denosumab within 90 days of bone metastasis (BM) diagnosis, and 86.8% were persistent at 3 months. **b** Fifteen point nine percent and 27.4% of patients had a history of pain medication (analgesics) prior to enrollment and at 3 months after the first dose of denosumab, respectively; 70.1% initiated denosumab within 90 days of BM diagnosis, and 86.6% were persistent at 3 months. **c** Thirty-eight point three percent and 36.0% of patients had a history of pain medication (analgesics) prior to enrollment and at 3 months after the first dose of denosumab, respectively; 77.9% initiated denosumab within 90 days of BM diagnosis, and 70.3% were persistent at 3 months. **d** Thirty-three point nine percent and 40.7% of patients had a history of pain medication (analgesics) prior to enrollment and at 3 months after the first dose of denosumab, respectively; 64.4% initiated denosumab within 90 days of BM diagnosis, and 84.8% were persistent at 3 months. **e** Forty-three point five percent and 49.7% of patients had a history of pain medication (analgesics) prior to enrollment and at 3 months after the first dose of denosumab, respectively; 76.3% initiated denosumab within 90 days of BM diagnosis, and 71.9% were persistent at 3 months. *EQ-5D-5L* EuroQoL 5-Dimension 5-Level

Most patients with solid tumors and BMs reported “no problems” or “some problems” during 9 months of follow-up (Supplementary Table 3). At month 3, the proportion of patients with no pain or discomfort was 38.3% (breast cancer), 47.4% (prostate cancer), 33.8% (lung cancer), 7.7% (kidney cancer), and 20.4% (other types of cancer; Fig. 2). The proportion with no problems walking was 63.8% (breast cancer), 64.0% (prostate cancer), 60.3% (lung cancer), 35.7% (kidney cancer), and 50.0% (other types of cancer). The proportion who had no problems with self-care was 79.1% (breast cancer), 83.1% (prostate cancer), 69.1% (lung cancer), 66.7% (kidney cancer), and 77.4% (other types of cancer). The proportion with no problems performing usual activities was 54.3% (breast cancer), 67.6% (prostate cancer), 41.2% (lung cancer), 30.8% (kidney cancer), and 33.3% (other types of cancer).

### Pain medication

Prior to enrollment, 15.9% (prostate cancer) to 43.5% (other types of cancer) of patients had a history of pain medication, and 27.7% (prostate cancer) to 49.7% (kidney cancer) received pain medication at 3 months after the first dose of denosumab (Supplementary Table 4). Of patients who received pain medication (measured before enrollment and at 3 months post-initiation), the most frequent medications with the highest Analgesic Quantification Algorithm (AQA) score were nonopioid analgesics (AQA score of 1) and strong opioids (with ≤ 75 mg oral morphine equivalent per day corresponding to an AQA score of 3).

## Discussion

This observational study provides insights into the real-world administration of denosumab per routine clinical practice in a wide geographic area spanning six countries in Central and Eastern Europe. The ESPACOMP 3-component consensus taxonomy for medication adherence [6] allowed the quantification of both adherence and nonadherence with denosumab. To our knowledge, this is the first study that examined the implementation of denosumab based on actual dosing history, considering both regularity and consistency of dosing per the recommended use.

Guidelines recommend that BTAs are initiated as soon as BMs are diagnosed, to delay SREs and reduce complications from metastatic bone disease [2, 3]. However, these recommendations are not always followed in routine clinical practice. In a study from Germany assessing the implementation of clinical guidelines (ESMO 2014 and national specialist guidelines), 70% of physicians reported that they adhered completely to the guidelines [18]. In the present study, up to 81.2% of patients received denosumab within 90 days of BM diagnosis, which is in line with studies investigating the initiation of BTAs (within 3 months of BM diagnosis, where specified) [11, 12, 16, 19–21]. Late initiation (nonadherence) occurred in at least one-fifth and potentially up to one-third of patients. It should be noted that some patients (maximum ~ 10%) had received prior antiresorptive therapy at baseline, and less than one-quarter of patients across cancer types had one or more SRE prior to enrollment. Insights from other real-world studies also indicate wide variations in the proportion of patients initiating therapy and the timing of initiation [15, 16, 21–25]. Various factors may influence the decision to delay BTAs, including a recent BM diagnosis (no time to initiate), perceived low risk of bone complications, patient refusal, patient frailty, and risk of osteonecrosis of the jaw [16, 21, 24]. Access and reimbursement, experience of the multidisciplinary team, and variations in recommendations for different cancer types may also influence the decision to delay treatment [16, 24]. The impact of delayed treatment with denosumab is not well studied. However, given the established evidence of the efficacy of denosumab in preventing SREs [26], there remains a need to align with guideline recommendations on timely initiation after BM diagnosis.

After initiation, it is important to understand whether patients receive denosumab regularly as prescribed. In our study, up to 72.5% of patients received regular and consistent dosing. Around one-third had longer than recommended gaps between administrations. The reasons for these gaps were not recorded but we suspect that this observed deviation from recommendations may have been due to a wider range of reasons, including clinical events occurring between the administrations (e.g., planned surgery or hospitalization), patients missing an appointment or difficulty attending the clinic (hospital setting), patient-physician decision or patient choice, access issues, a perception that osteoprotection is not an important aspect of cancer treatment, delays in dental treatment, or a lack of physician BTA experience. The impact of suboptimal implementation of denosumab on clinical outcomes is not clear; contradictory results have been reported in the few studies that examined the effect on SREs when de-escalating denosumab dosing to every 12 weeks compared with the recommended every 4 weeks [27–33]. The pharmacokinetics of denosumab support regular and consistent dosing; denosumab has a half-life of 28 days [34], and ESMO guidelines state that “unlike bisphosphates, denosumab is not stored in bone and interrupting its administration is probably not without risk. Based on its pharmacodynamics and systemic distribution, continuous monthly therapy with denosumab should be adhered to until shown otherwise” [2]. Suboptimal implementation may, therefore, impact the efficacy of denosumab.

Most patients with solid tumors and BMs in our analysis were persistent with denosumab (no gap > 60 days) at 3 months; the proportion of persistent patients reduced over time across all cancer types. Persistence with denosumab has been previously described; however, the terminology and analytic approaches used have not been consistent [6, 8, 11–13]. Therefore, making comparisons between our findings and previous evidence should be done with caution. Further research is needed to understand the observed variations in persistence between studies and cancer types.

In the present cohort, the cumulative incidence of death exceeded the cumulative incidence of non-persistence with denosumab and, therefore, was competing with it. Consequently, a Kaplan–Meier analysis that would require either censoring patients who died or combining both types of events into a composite endpoint would yield distorted estimates for the probability of remaining persistent [17]. As such, competing risk analysis provides more accurate estimates; hence, the cumulative incidences for both death and non-persistence were presented. The overall level of non-persistence to denosumab was low in patients with solid tumors and BMs.

Despite adherence with denosumab being generally high across the initiation, implementation, and persistence phases, there is scope for adherence to be improved. Some potential strategies are summarized in Table 3 [35–38]. Strategies to improve medication adherence may reduce avoidable hospitalizations and improve patient quality of life, with resulting cost savings [39, 40].

**Table 3:** Clinical recommendations for improving medication adherence with denosumab based on the Action, Actor, Context, Target, Time (AACTT) framework [35]

Strategy for improving medication adherence	Target (patient/physician)	Individual or group actioning the strategy	Timing of strategy
Initiation of denosumab immediately after BM diagnosis	Patient	Physician	Immediately after BM diagnosis
Supplementation with calcium and vitamin D to reduce the risk of hypocalcemia	Patient	Physician/nurse	During denosumab treatment
Reminders for appointments (e.g., diaries or text messages)	Patient	Clinic support staff	During denosumab treatment
Education on ESMO guidelines, tailored to the specialty of the physician and depending on experience	Physician	Physician/nurse	Ongoing
Education on SREs to address gaps in bone health education [36] and encourage patients to prioritize appointments for BTAs	Patient	Physician/nurse	Prior to denosumab initiation/during denosumab treatment
Information to address gaps (e.g., in the form of documents, videos, or websites) [37]	Patient	Physician/nurse	Prior to denosumab initiation
Facilitation of communication between patients and their physician [37]	Patient/physician	Patient/physician/nurse	Ongoing
Consider off-site, drive-through, or home administration of denosumab, where feasible, if adherence is disrupted (e.g., due to the COVID-19 pandemic) in patients receiving denosumab [38]	Patient	Physician	During denosumab treatment

*BM* bone metastasis, *BTA* bone-targeting agent, *COVID-19* coronavirus disease 2019, *ESMO* European Society for Medical Oncology, *SRE* skeletal-related event

It was not possible to evaluate PROs or pain according to adherence versus nonadherence in our analysis. PRO results varied across cancer types; most patients reported “no problems” or “some problems” with pain, mobility, self-care, and usual activities throughout the study. Few patients received the strongest opioids in our study; however, pain medication data should be interpreted with caution as data were not available for each instance of PRO questionnaire completion and longer observation times are needed.

For continuous monthly treatment of denosumab, even when initiated and implemented according to guidelines, it is important to consider the risk of adverse events (AEs); the most important AE associated with prolonged BTA use is osteonecrosis of the jaw, with an incidence of 1% per year on BTA treatment [2]. Dental examination evaluation, therefore, is recommended before initiation of denosumab. No new safety signals were identified in the individual studies reported here [4, 9]. As such, no further safety analyses were performed.

The analysis has several strengths. Centers were selected based on a balanced distribution of sites with regard to geography and specialty, combined with consecutive enrollment of patients. As such, they provided a patient sample similar to a typical population of patients with solid tumors and BMs in similar healthcare settings. Combining two studies allowed adherence to be assessed across many patients over a sizable European geographic region. Additionally, we used the 3-component consensus taxonomy for describing medication adherence as recommended by ESPACOMP [6], with some adaptations (Supplementary Table 1), which is a robust method for defining medication adherence.

Nevertheless, this analysis also has limitations. Selection bias might have occurred as the inclusion of centers, physicians, and patients was likely influenced by willingness to participate, and the data could potentially overrepresent patients who frequently visit clinics for care. Accordingly, it is difficult to compare adherence from real-world studies with those from clinical trials where patients are under controlled settings. Country heterogeneity and treatment variations also likely exist. Thus, results may not be generalizable to all patients with solid tumors and BMs. Importantly, no data on SREs were collected in the individual studies, and patients were not followed up in the long term. As a result, it is not possible to draw any conclusions regarding the impact of medication adherence/nonadherence (including long-term treatment) on the incidence of SREs or other clinical outcomes. In addition, a diagnosis of SRE at enrollment may have had an impact on the urgency of starting bone-modifying therapy, compared with an incidentally found or asymptomatic bone metastasis. Finally, no information was available on the reasons for initiation or discontinuation of denosumab, and data on renal impairment, hypocalcemia/hypercalcemia, prior calcemia, and bone pain at enrollment were unavailable for most patients.

This study used a 3-component consensus taxonomy for defining medication adherence in patients receiving denosumab. This taxonomy provides clear, robust, and consistent measures of three key aspects of medication adherence and nonadherence. In this study, this approach provides valuable insights into medication adherence with denosumab in patients with solid tumors and BMs when treated as per routine clinical practice in their respective countries, but the study limitations mean that this reflects only a subset of the overall patient population. Therefore, further studies using the same 3-component consensus taxonomy are worth pursuing.

## Conclusions

We described three phases of medication adherence in patients with solid tumors and BMs who received denosumab in routine clinical practice. Most patients experienced timely initiation and optimal implementation. There were variations by cancer type in the initiation, implementation, and persistence of denosumab. Further studies are needed to examine determinants of medication adherence and nonadherence, and to improve adherence with denosumab.

## Supplementary Information

Below is the link to the electronic supplementary material.

Supplementary file1 (PDF 1105 KB)

## Data Availability

Qualified researchers may request data from Amgen clinical studies. Complete details are available at the following: https://www.amgen.com/science/clinical-trials/clinical-data-transparency-practices/clinical-trial-data-sharing-request/.

## Code availability

N/A
