# Supplementary material for: Medication adherence with denosumab in patients with bone metastases from solid tumors treated in routine clinical settings: a retrospective study
Source: Support Care Cancer. 2022 Sep 6;30(11):9267–78. doi: 10.1007/s00520-022-07333-7 (PMC9446633; doi:10.1007/s00520-022-07333-7)
Supplement: Supplementary file 1 — Supplementary file1 (PDF 1105 KB) [file 520_2022_7333_MOESM1_ESM.pdf]

Supportive Care in Cancer

Supplementary material to:

Medication adherence with denosumab in patients with bone metastases from solid tumors treated in routine clinical settings: a retrospective study

Ingo J. Diel[1], Richard Greil[2,3], Jan Janssen[4], Christian W. Kluike[5], Bagmeet Behera[6], Ali Abbasi[7,*], Anouchka Seesaghur[7], Michael Kellner[8,*], Christine Jaeger[9], Katja Bjorklof[10], Antoaneta Tomova[11], Ferdinand Haslbauer[12]

[1]Praxisklinik am Rosengarten, Mannheim, Germany; [2]Paracelsus Medizinische Privatuniversität, Salzburg, Austria; [3]Salzburg Cancer Research Institute-Center for Clinical Cancer and Immunology Trials and Cancer Cluster Salzburg, Austria; [4]Medizinische Studiengesellschaft Nord-West GmbH, Westerstede, Germany; [5]Praxis für Urologie Kluike und Weiler, Lüneburg, Germany; [6]Amgen Research Munich GmbH, Munich, Germany; [7]Center for Observational Research, Amgen, Uxbridge, UK; [8]Amgen GmbH, Munich, Germany; [9]Amgen GmbH, Vienna, Austria; [10]Amgen Europe GmbH, Rotkreuz, Switzerland; [11]Complex Oncology Center Plovdiv EOOD, Plovdiv, Bulgaria; [12]Salzkammergut Klinikum Vöcklabruck, Vöcklabruck, Austria

*Affiliation at the time the research was conducted

Corresponding author:
Professor Ingo J. Diel
Email: ingodiel@gmail.com

CONTENTS

Supplementary Table 1 Methods used to assess each phase of medication adherence with denosumab, based on ESPACOMP recommendations

Supplementary Table 2 Patient comorbidities at baseline by cancer type

Supplementary Table 3 Patient-reported outcomes at months 0, 3, 6, and 9 of denosumab initiation via the EQ-5D-5L questionnaire domains by cancer type

Supplementary Table 4 Pain medication use prior to enrollment and at 3 months after denosumab initiation

Supplementary Fig. 1 X-TREME study and Study 240: study design

Supplementary Fig. 2 Cumulative risk of death during follow-up and risk of non-persistence in patients who received denosumab and who had **a** breast cancer, **b** prostate cancer, **c** lung cancer, **d** kidney cancer, or **e** other cancer types

Supplementary Fig. 3 Cumulative risk of non-persistence

Supplementary Fig. 4 Cumulative risk of non-persistence and death (breast cancer)

References

Supplementary Table 1 Methods used to assess each phase of medication adherence with denosumab, based on ESPACOMP recommendations [1]

Phase of medication adherence		Denosumab exposure for each patient	Description	Groups of interest
1	Initiation	Based on first-ever denosumab administration	All patients have started on denosumab. Alignment of denosumab initiation with ESMO guidelines [2], based on the recommendation to initiate BTA treatment as soon as BM is detected. Time to initiation was defined as time from BM diagnosis to the record of the first-ever denosumab administration	1. Timely initiation: denosumab initiation ≤90 days of BM diagnosis<br>2. Late initiation: denosumab initiation >90 days of BM diagnosis
2	Implementation	Based on the patient's full dosing history (each denosumab administration for patients with ≥3 doses of denosumab)	Patients classified according to extent to which gaps between administrations correspond to the recommended administration gap of 28 days, in terms of both regularity and consistency<br>$Regularity = \frac{\text{28 days}}{Mean\ dose\ gap\ (in\ days)}$<br>$Inconsistency = Error(Regularity)$<br>Regular implementation occurs when the mean dose gap is 28 days ± 2.8 days (i.e., 10% deviation). Consistent implementation occurs when there are no large deviations from the mean dose gap (i.e., inconsistency ≤0.2)	1. Optimal implementation: regular and consistent dosing (regularity ≥0.9 and ≤1.1 and inconsistency ≤0.2)<br>2. Suboptimal implementation: irregular or inconsistent dosing (regularity <0.9 or >1.1 and inconsistency >0.2)
3	Persistence	Based on the first-ever denosumab and last on-study denosumab administrations	Defined as the time from the date of the first-ever denosumab to the discontinuation date (last record of denosumab administration before a gap >60 days between consecutive denosumab administrations), lost to follow-up, date of switch to another therapy, or end of the study period; estimated at different time points	1. Persistent with denosumab at 3, 6, 9, or 12 months<br>2. Non-persistent[a,b] with denosumab at 3, 6, 9, or 12 months

BM bone metastasis, *BTA* bone-targeted agent, *ESMO* European Society for Medical Oncology, *ESPACOMP* European Society for Patient Adherence, COMpliance, and Persistence

[a] Patients were defined as non-persistent if they refused to receive further denosumab treatment, the physician stopped treatment, treatment was discontinued due to a reportable adverse reaction, or the patient was lost to follow-up

[b] Patients were censored if the censoring day was before time to non-persistence if: they died, finished treatment per study protocol after 52 weeks, withdrew consent, or were lost to follow-up

Supplementary Table 2 Patient comorbidities at baseline by cancer type

	**Breast cancer**	**Prostate cancer**	**Lung cancer**	**Kidney cancer**	**Other types of cancer**
Patients with bone metastases, *n*	842	441	222	59	177
Comorbidities prior to/at enrollment					
Heart attack	3 (0.4)	21 (4.8)	11 (5.0)	2 (3.4)	3 (1.7)
Congestive heart failure	8 (0.9)	20 (4.5)	7 (3.2)	0 (0.0)	7 (4.0)
Peripheral arterial disease	8 (0.9)	20 (4.5)	17 (7.7)	3 (5.1)	11 (6.2)
Cerebrovascular disease	9 (1.1)	9 (2.0)	6 (2.7)	0 (0.0)	3 (1.7)
Dementia	1 (0.1)	3 (0.7)	0 (0.0)	1 (1.7)	0 (0.0)
Chronic lung disease	17 (2.0)	20 (4.5)	51 (23.0)	3 (5.1)	11 (6.2)
Connective tissue disease	2 (0.2)	0 (0.0)	0 (0.0)	0 (0.0)	0 (0.0)
Ulcer disease	3 (0.4)	0 (0.0)	5 (2.3)	0 (0.0)	2 (1.1)
Mild liver disease	8 (0.9)	7 (1.6)	6 (2.7)	1 (1.7)	8 (4.5)
Moderate to severe liver disease	9 (1.1)	4 (0.9)	4 (1.8)	0 (0.0)	3 (1.7)
Diabetes	74 (8.7)	55 (12.5)	32 (14.4)	4 (6.8)	26 (14.7)
Diabetes with end-organ damage	9 (1.1)	0 (0.0)	3 (1.4)	1 (1.7)	3 (1.7)
Hemiplegia	1 (0.1)	0 (0.0)	1 (0.5)	0 (0.0)	0 (0.0)
Moderate to severe kidney disease	33 (3.9)	35 (7.9)	15 (6.8)	18 (30.5)	12 (6.8)

	Breast cancer	Prostate cancer	Lung cancer	Kidney cancer	Other types of cancer
Tumor	511 (60.2)	295 (66.9)	159 (71.6)	50 (84.7)	116 (65.5)
Leukemia	1 (0.1)	0 (0.0)	1 (0.5)	3 (5.1)	0 (0.0)
Lymphoma	1 (0.1)	8 (1.8)	1 (0.5)	3 (5.1)	0 (0.0)
AIDS	0 (0.0)	0 (0.0)	0 (0.0)	0 (0.0)	1 (0.6)

AIDS acquired immunodeficiency syndrome

Data shown as *n* (%) unless indicated otherwise

Supplementary Table 3 Patient-reported outcomes at months 0, 3, 6, and 9 of denosumab initiation via the EQ-5D-5L questionnaire domains by cancer type

Characteristic	Month 0 ± 5 weeks	Month 3 ± 5 weeks	Month 6 ± 5 weeks	Month 9 ± 5 weeks
**Breast cancer**	**All**			
**EQ5DPAIN – Pain**				
Number	483	282	253	290
Mean score	1.78	1.68	1.63	1.65
Standard deviation	0.62	0.59	0.57	0.59
Median score	2	2	2	2
Q1 score	1	1	1	1
Q3 score	2	2	2	2
Minimum score	1	1	1	1
Maximum score	4	3	3	3
No pain or discomfort, *n* (%)	157 (32.5)	108 (38.3)	104 (41.1)	119 (41.0)
Mild pain or discomfort, *n* (%)	278 (57.6)	156 (55.3)	138 (54.5)	154 (53.1)
Moderate pain or discomfort, *n* (%)	46 (9.5)	18 (6.4)	11 (4.3)	17 (5.9)
Severe pain or discomfort, *n* (%)	2 (0.4)	0 (0.0)	0 (0.0)	0 (0.0)
**EQ5DMOB – Mobility**				
Number	482	282	254	289
Mean score	1.39	1.38	1.37	1.35
Standard deviation	0.51	0.52	0.51	0.49
Median score	1	1	1	1
Q1 score	1	1	1	1
Q3 score	2	2	2	2
Minimum score	1	1	1	1
Maximum score	3	3	3	3
No problem in walking about, *n* (%)	298 (61.8)	180 (63.8)	162 (63.8)	191 (66.1)
Some problems in walking about, *n* (%)	179 (37.1)	97 (34.4)	89 (35.0)	96 (33.2)
Confined to bed, *n* (%)	5 (1.0)	5 (1.8)	3 (1.2)	2 (0.7)

Characteristic	Month 0 ± 5 weeks	Month 3 ± 5 weeks	Month 6 ± 5 weeks	Month 9 ± 5 weeks
**EQ5DSC – Self-Care**				
Number	483	282	253	289
Mean score	1.26	1.24	1.23	1.21
Standard deviation	0.50	0.49	0.48	0.46
Median score	1	1	1	1
Q1 score	1	1	1	1
Q3 score	1	1	1	1
Minimum score	1	1	1	1
Maximum score	3	3	3	3
No problem with self-care, *n* (%)	369 (76.4)	223 (79.1)	201 (79.4)	235 (81.3)
Some problems washing or dressing themselves, *n* (%)	101 (20.9)	51 (18.1)	45 (17.8)	47 (16.3)
Unable to wash or dress themselves, *n* (%)	13 (2.7)	8 (2.8)	7 (2.8)	7 (2.4)
**EQ5DDACT – Usual Activities**				
Number	483	282	254	290
Mean score	1.55	1.51	1.47	1.44
Standard deviation	0.61	0.60	0.59	0.61
Median score	1	1	1	1
Q1 score	1	1	1	1
Q3 score	2	2	2	2
Minimum score	1	1	1	1
Maximum score	3	3	3	3
No problems with performing usual activities, *n* (%)	248 (51.3)	153 (54.3)	148 (58.3)	180 (62.1)
Some problems performing usual activities, *n* (%)	205 (42.4)	113 (40.1)	93 (36.6)	92 (31.7)
Unable to perform usual activities, *n* (%)	30 (6.2)	16 (5.7)	13 (5.1)	18 (6.2)

**Characteristic**	**Month 0 ± 5 weeks**	**Month 3 ± 5 weeks**	**Month 6 ± 5 weeks**	**Month 9 ± 5 weeks**
**Prostate cancer**	**All**			
**EQ5DPAIN – Pain**				
Number	279	137	138	196
Mean score	1.63	1.57	1.51	1.66
Standard deviation	0.57	0.58	0.57	0.57
Median score	2	2	1	2
Q1 score	1	1	1	1
Q3 score	2	2	2	2
Minimum score	1	1	1	1
Maximum score	3	3	3	4
No pain or discomfort, *n* (%)	116 (41.6)	65 (47.4)	72 (52.2)	76 (38.8)
Mild pain or discomfort, *n* (%)	150 (53.8)	66 (48.2)	61 (44.2)	112 (57.1)
Moderate pain or discomfort, *n* (%)	13 (4.7)	6 (4.4)	5 (3.6)	7 (3.6)
Severe pain or discomfort, *n* (%)	0 (0.0)	0 (0.0)	0 (0.0)	1 (0.5)
**EQ5DMOB – Mobility**				
Number	280	136	138	196
Mean score	1.42	1.36	1.36	1.43
Standard deviation	0.51	0.48	0.48	0.51
Median score	1	1	1	1
Q1 score	1	1	1	1
Q3 score	2	2	2	2
Minimum score	1	1	1	1
Maximum score	3	2	2	3
No problem in walking about, *n* (%)	164 (58.6)	87 (64.0)	88 (63.8)	112 (57.1)
Some problems in walking about, *n* (%)	114 (40.7)	49 (36.0)	50 (36.2)	83 (42.3)
Confined to bed, *n* (%)	2 (0.7)	0 (0.0)	0 (0.0)	1 (0.5)

Characteristic	Month 0 ± 5 weeks	Month 3 ± 5 weeks	Month 6 ± 5 weeks	Month 9 ± 5 weeks
**EQ5DSC – Self-Care**				
Number	280	136	137	196
Mean score	1.26	1.18	1.20	1.26
Standard deviation	0.48	0.43	0.42	0.48
Median score	1	1	1	1
Q1 score	1	1	1	1
Q3 score	1	1	1	1
Minimum score	1	1	1	1
Maximum score	3	3	3	3
No problem with self-care, *n* (%)	214 (76.4)	113 (83.1)	111 (81.0)	149 (76.0)
Some problems washing or dressing themselves, *n* (%)	60 (21.4)	21 (15.4)	25 (18.2)	43 (21.9)
Unable to wash or dress themselves, *n* (%)	6 (2.1)	2 (1.5)	1 (0.7)	4 (2.0)
	280 (100.0)	136 (100.0)	137 (100.0)	196 (100.0)
**EQ5DDACT – Usual Activities**				
Number	279	136	137	195
Mean score	1.44	1.37	1.31	1.45
Standard deviation	0.57	0.57	0.50	0.58
Median score	1	1	1	1
Q1 score	1	1	1	1
Q3 score	2	2	2	2
Minimum score	1	1	1	1
Maximum score	3	3	3	3
No problems with performing usual activities, *n* (%)	167 (59.9)	92 (67.6)	96 (70.1)	117 (60.0)
Some problems performing usual activities, *n* (%)	101 (36.2)	38 (27.9)	39 (28.5)	69 (35.4)
Unable to perform usual activities, *n* (%)	11 (3.9)	6 (4.4)	2 (1.5)	9 (4.6)

Characteristic	Month 0 ± 5 weeks	Month 3 ± 5 weeks	Month 6 ± 5 weeks	Month 9 ± 5 weeks
**Lung cancer**	**All**			
**EQ5DPAIN – Pain**				
Number	130	68	38	40
Mean score	1.85	1.76	1.71	1.58
Standard deviation	0.58	0.63	0.65	0.59
Median score	2	2	2	2
Q1 score	2	1	1	1
Q3 score	2	2	2	2
Minimum score	1	1	1	1
Maximum score	4	3	3	3
No pain or discomfort, *n* (%)	32 (24.6)	23 (33.8)	15 (39.5)	19 (47.5)
Mild pain or discomfort, *n* (%)	87 (66.9)	38 (55.9)	19 (50.0)	19 (47.5)
Moderate pain or discomfort, *n* (%)	10 (7.7)	7 (10.3)	4 (10.5)	2 (5)
Severe pain or discomfort, *n* (%)	1 (0.8)	0 (0.0)	0 (0.0)	0 (0.0)
**EQ5DMOB – Mobility**				
Number	130	68	38	40
Mean score	1.51	1.40	1.42	1.40
Standard deviation	0.53	0.49	0.60	0.50
Median score	1	1	1	1
Q1 score	1	1	1	1
Q3 score	2	2	2	2
Minimum score	1	1	1	1
Maximum score	3	2	3	2
No problem in walking about, *n* (%)	66 (50.8)	41 (60.3)	24 (63.2)	24 (60.0)
Some problems in walking about, *n* (%)	62 (47.7)	27 (39.7)	12 (31.6)	16 (40.0)
Confined to bed, *n* (%)	2 (1.5)	0 (0.0)	2 (5.3)	0 (0.0)

Characteristic	Month 0 ± 5 weeks	Month 3 ± 5 weeks	Month 6 ± 5 weeks	Month 9 ± 5 weeks
**EQ5DSC – Self-Care**				
Number	130	68	38	40
Mean score	1.35	1.34	1.34	1.45
Standard deviation	0.55	0.54	0.58	0.64
Median score	1	1	1	1
Q1 score	1	1	1	1
Q3 score	2	2	2	2
Minimum score	1	1	1	1
Maximum score	3	3	3	
No problem with self-care, *n* (%)	89 (68.5)	47 (69.1)	27 (71.1)	25 (62.5)
Some problems washing or dressing themselves, *n* (%)	36 (27.7)	19 (27.9)	9 (23.7)	12 (30.0)
Unable to wash or dress themselves, *n* (%)	5 (3.8)	2 (2.9)	2 (5.3)	3 (7.5)
**EQ5DDACT – Usual Activities**				
Number	130	68	38	40
Mean score	1.74	1.68	1.71	1.68
Standard deviation	0.68	0.63	0.69	0.73
Median score	2	2	2	2
Q1 score	1	1	1	1
Q3 score	2	2	2	2
Minimum score	1	1	1	1
Maximum score	3	3	3	3
No problems with performing usual activities, *n* (%)	51 (39.2)	28 (41.1)	16 (42.1)	19 (47.5)
Some problems performing usual activities, *n* (%)	62 (47.7)	34 (50.0)	17 (44.7)	15 (37.5)
Unable to perform usual activities, *n* (%)	17 (13.1)	6 (8.8)	5 (13.2)	6 (15.0)

Characteristic	Month 0 ± 5 weeks	Month 3 ± 5 weeks	Month 6 ± 5 weeks	Month 9 ± 5 weeks
**Kidney cancer**	**All**			
**EQ5DPAIN – Pain**				
Number	40	13	11	12
Mean score	1.85	1.92	2.00	2.17
Standard deviation	0.48	0.28	0.45	0.58
Median score	2	2	2	2
Q1 score	2	2	2	2
Q3 score	2	2	2	2.25
Minimum score	1	1	1	1
Maximum score	3	2	3	3
No pain or discomfort, *n* (%)	8 (20.0)	1 (7.7)	1 (9.1)	1 (8.3)
Mild pain or discomfort, *n* (%)	30 (75.0)	12 (92.3)	9 (81.8)	8 (66.7)
Moderate pain or discomfort, *n* (%)	2 (5.0)	0 (0.0)	1 (9.1)	3 (25.0)
Severe pain or discomfort, *n* (%)	0 (0.0)	0 (0.0)	0 (0.0)	0 (0.0)
**EQ5DMOB – Mobility**				
Number	40	14	11	12
Mean score	1.53	1.64	1.45	1.42
Standard deviation	0.51	0.50	0.52	0.51
Median score	2	2	1	1
Q1 score	1	1	1	1
Q3 score	2	2	2	2
Minimum score	1	1	1	1
Maximum score	2	2	2	2
No problem in walking about, *n* (%)	19 (47.5)	5 (35.7)	6 (54.5)	7 (58.3)
Some problems in walking about, *n* (%)	21 (52.5)	9 (64.3)	5 (45.5)	5 (41.7)
Confined to bed, *n* (%)	0 (0.0)	0 (0.0)	0 (0.0)	0 (0.0)

Characteristic	Month 0 ± 5 weeks	Month 3 ± 5 weeks	Month 6 ± 5 weeks	Month 9 ± 5 weeks
**EQ5DSC – Self-Care**				
Number	40	12	11	12
Mean score	1.25	1.33	1.27	1.42
Standard deviation	0.44	0.49	0.47	0.67
Median score	1	1	1	1
Q1 score	1	1	1	1
Q3 score	1.25	2	1.5	2
Minimum score	1	1	1	1
Maximum score	2	2	2	3
No problem with self-care, *n* (%)	30 (75.0)	8 (66.7)	8 (72.7)	8 (66.7)
Some problems washing or dressing themselves, *n* (%)	10 (25.0)	4 (33.3)	3 (27.3)	3 (25.0)
Unable to wash or dress themselves, *n* (%)	0 (0.0)	0 (0.0)	0 (0.0)	1 (8.3)
**EQ5DDACT – Usual Activities**				
Number	40	13	11	11
Mean score	1.65	1.77	2.00	1.82
Standard deviation	0.58	0.60	0.63	0.60
Median score	2	2	2	2
Q1 score	1	1	2	1.5
Q3 score	2	2	2	2
Minimum score	1	1	1	1
Maximum score	3	3	3	3
No problems with performing usual activities, *n* (%)	16 (40.0)	4 (30.8)	2 (18.2)	3 (27.3)
Some problems performing usual activities, *n* (%)	22 (55.0)	8 (61.5)	7 (63.6)	7 (63.6)
Unable to perform usual activities, *n* (%)	2 (5.0)	1 (7.7)	2 (18.2)	1 (9.1)

**Characteristic**	**Month 0 ± 5 weeks**	**Month 3 ± 5 weeks**	**Month 6 ± 5 weeks**	**Month 9 ± 5 weeks**
**Other types of cancer**	**All**			
**EQ5DPAIN – Pain**				
Number	100	54	30	32
Mean score	1.85	1.89	1.77	1.78
Standard deviation	0.48	0.54	0.50	0.49
Median score	2	2	2	2
Q1 score	2	2	1.25	1.75
Q3 score	2	2	2	2
Minimum score	1	1	1	1
Maximum score	3	3	3	3
No pain or discomfort, *n* (%)	20 (20.0)	11 (20.4)	8 (26.7)	8 (25.0)
Mild pain or discomfort, *n* (%)	75 (75.0)	38 (70.4)	21 (70.0)	23 (71.9)
Moderate pain or discomfort, *n* (%)	5 (5.0)	5 (9.3)	1 (3.3)	1 (3.1)
Severe pain or discomfort, *n* (%)	0 (0.0)	0 (0.0)	0 (0.0)	0 (0.0)
**EQ5DMOB – Mobility**				
Number	100	54	30	32
Mean score	1.42	1.54	1.37	1.66
Standard deviation	0.52	0.57	0.56	0.65
Median score	1	1.5	1	2
Q1 score	1	1	1	1
Q3 score	2	2	2	2
Minimum score	1	1	1	1
Maximum score	3	3	3	3
No problem in walking about, *n* (%)	59 (59.0)	27 (50.0)	20 (66.7)	14 (43.8)
Some problems in walking about, *n* (%)	40 (40.0)	25 (46.3)	9 (30.0)	15 (46.9)
Confined to bed, *n* (%)	1 (1.0)	2 (3.7)	1 (3.3)	3 (9.4)

Characteristic	Month 0 ± 5 weeks	Month 3 ± 5 weeks	Month 6 ± 5 weeks	Month 9 ± 5 weeks
**EQ5DSC – Self-Care**				
Number	100	53	30	32
Mean score	1.26	1.30	1.37	1.53
Standard deviation	0.50	0.61	0.61	0.67
Median score	1	1	1	1
Q1 score	1	1	1	1
Q3 score	1	1	2	2
Minimum score	1	1	1	1
Maximum score	3	3	3	3
No problem with self-care, *n* (%)	77 (77.0)	41 (77.4)	21 (70.0)	18 (56.3)
Some problems washing or dressing themselves, *n* (%)	20 (20.0)	8 (15.1)	7 (23.3)	11 (34.4)
Unable to wash or dress themselves, *n* (%)	3 (3.0)	4 (7.5)	2 (6.7)	3 (9.4)
**EQ5DDACT – Usual Activities**				
Number	100	54	30	32
Mean score	1.65	1.76	1.5	1.78
Standard deviation	0.63	0.61	0.57	0.75
Median score	2	2	1	2
Q1 score	1	1	1	1
Q3 score	2	2	2	2
Minimum score	1	1	1	1
Maximum score	3	3	3	3
No problems with performing usual activities, *n* (%)	43 (43.0)	18 (33.3)	16 (53.3)	13 (40.6)
Some problems performing usual activities, *n* (%)	49 (49.0)	31 (57.4)	13 (43.3)	13 (40.6)
Unable to perform usual activities, *n* (%)	8 (8.0)	5 (9.3)	1 (3.3)	6 (18.8)

EQ-5D-5L EuroQol 5-Dimension 5-Level, *PRO* patient-reported outcome

PROs were measured using the EQ-5D-5L questionnaire; each domain (pain, mobility, self-care, and usual activities) were rated on a 1–5 scale (1 corresponding to no problems, 5 corresponding to extreme problems)

Supplementary Table 4 Pain medication use prior to enrollment and at 3 months after denosumab initiation

Pain medication use	Breast cancer	Prostate cancer	Lung cancer	Kidney cancer	Other types of cancer
Patients, *n*	842	441	222	59	177
Patients with history of pain medication prior to enrollment					
Yes	191 (22.7)	70 (15.9)	85 (38.3)	20 (33.9)	77 (43.5)
No	651 (77.3)	371 (84.1)	137 (61.7)	39 (66.1)	100 (56.5)
Pain medication classification in patients with a history of pain medication prior to enrollment					
Patients, *n*	191	70	85	20	77
AQA undefined[a]	1 (0.5)	0 (0.0)	0 (0.0)	0 (0.0)	2 (2.6)
Nonopioid analgesics	95 (49.7)	41 (58.6)	34 (40.0)	9 (45.0)	25 (32.5)
Weak opioids[b]	22 (11.5)	12 (17.1)	13 (15.3)	3 (15.0)	12 (15.6)
Strong opioids ≤75 mg OME/day	59 (30.9)	14 (20.0)	29 (34.1)	7 (35.0)	30 (39.0)
Strong opioids >75–150 mg OME/day	11 (5.8)	3 (4.3)	4 (4.7)	0 (0.0)	4 (5.2)
Strong opioids >150–300 mg OME/day	2 (1.0)	0 (0.0)	2 (2.4)	0 (0.0)	2 (2.6)
Strong opioids >300–600 mg OME/day	1 (0.5)	0 (0.0)	3 (3.5)	0 (0.0)	1 (1.3)
Strong opioids >600 mg OME/day	0 (0.0)	0 (0.0)	0 (0.0)	1 (5.0)	1 (1.3)
Pain medication at 3 months after denosumab initiation					

**Pain medication use**	**Breast cancer**	**Prostate cancer**	**Lung cancer**	**Kidney cancer**	**Other types of cancer**
Yes	256 (30.4)	122 (27.7)	80 (36.0)	24 (40.7)	88 (49.7)
No	586 (69.6)	319 (72.3)	142 (64.0)	35 (59.3)	89 (50.3)
Pain medication classification in patients who had received pain medication at 3 months after denosumab initiation					
Patients, *n*	256	122	80	24	88
AQA undefined[a]	2 (0.8)	0 (0.0)	0 (0.0)	0 (0.0)	1 (1.1)
Nonopioid analgesics	128 (50.0)	73 (59.8)	22 (27.5)	11 (45.8)	29 (33.0)
Weak opioids[b]	33 (12.9)	22 (18.0)	15 (18.8)	0 (0.0)	11 (12.5)
Strong opioids ≤75 mg OME/day	76 (29.7)	24 (19.7)	33 (41.3)	9 (37.5)	35 (39.8)
Strong opioids >75–150 mg OME/day	12 (4.7)	3 (2.5)	6 (7.5)	3 (12.5)	5 (5.7)
Strong opioids >150–300 mg OME/day	3 (1.2)	0 (0.0)	2 (2.5)	0 (0.0)	3 (3.4)
Strong opioids >300–600 mg OME/day	2 (0.8)	0 (0.0)	2 (2.5)	1 (4.2)	3 (3.4)
Strong opioids >600 mg OME/day	0 (0.0)	0 (0.0)	0 (0.0)	0 (0.0)	1 (1.1)

AQA Analgesic Quantification Algorithm, *OME* oral morphine equivalent

Data shown as *n* (%) unless indicated otherwise

[a] For certain drugs, for example, fentanyl, tilidin

[b] For example, meperidine, codeine, tramadol

Supplementary Fig. 1 X-TREME study and Study 240: study design [3, 4]

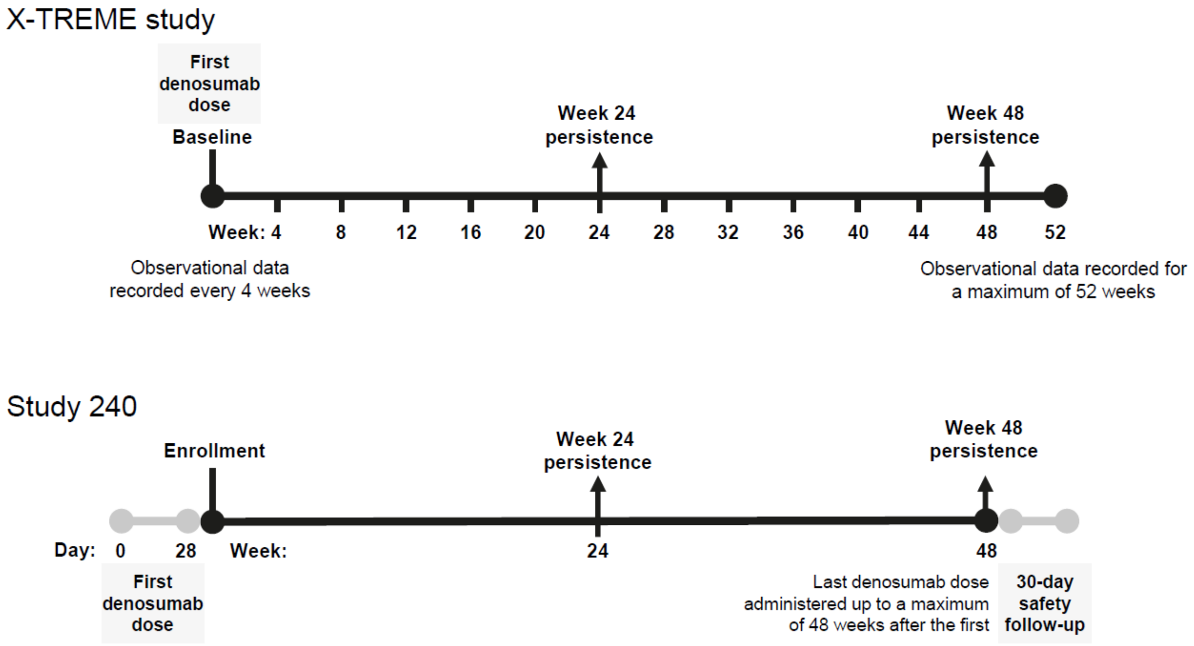

Supplementary Fig. 2 Cumulative risk of death during follow-up and risk of non-persistence in patients who received denosumab and who had **a** breast cancer, **b** prostate cancer, **c** lung cancer, **d** kidney cancer, or **e** other cancer types. Scales are not uniform for each figure panel. Solid lines represent cumulative incidence; surrounding shaded areas represent 95% confidence intervals

a

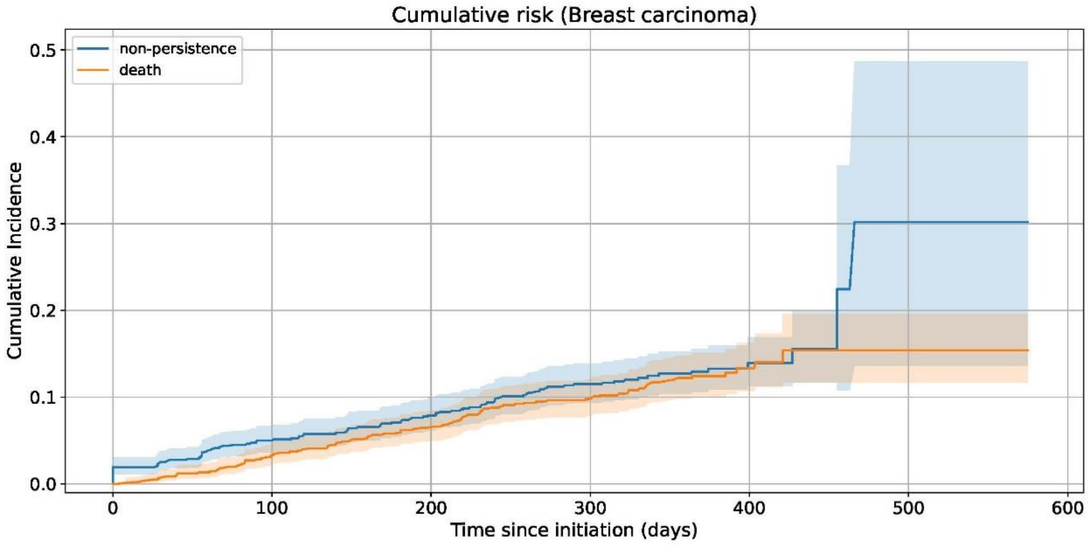

Event Counts

At Risk	816.0	721.0	631.0	537.0	103.0	3.0	0.0
Censored	10.0	53.0	97.0	141.0	551.0	646.0	649.0
Non-persistence	17.0	42.0	64.0	90.0	99.0	102.0	102.0
Death	0.0	27.0	51.0	75.0	90.0	92.0	92.0

b

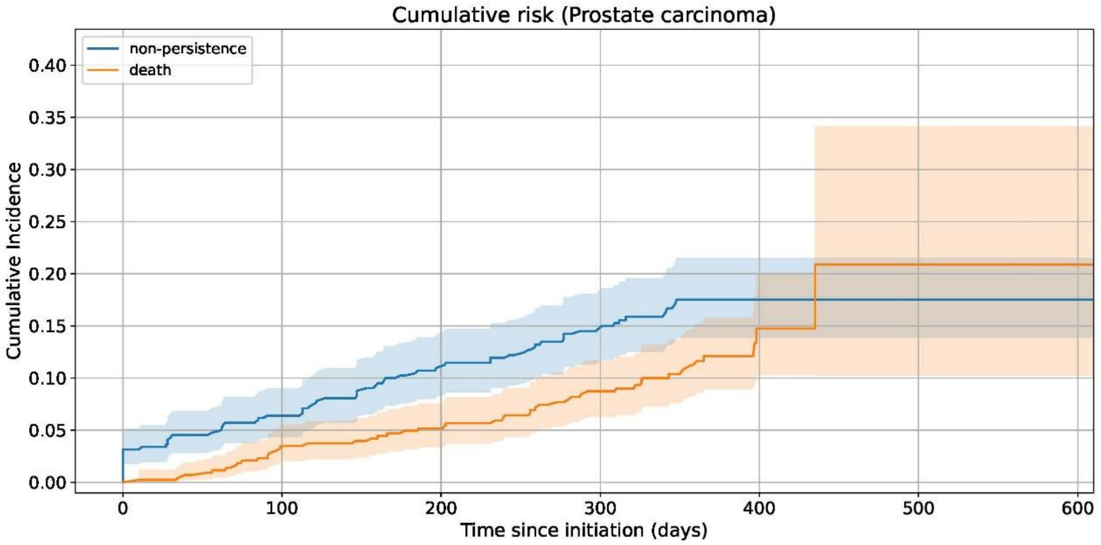

Event Counts

At Risk	426.0	381.0	340.0	298.0	44.0	2.0	1.0
Censored	5.0	21.0	36.0	49.0	284.0	325.0	326.0
Non-persistence	15.0	29.0	48.0	63.0	71.0	71.0	71.0
Death	0.0	15.0	22.0	36.0	47.0	48.0	48.0

c

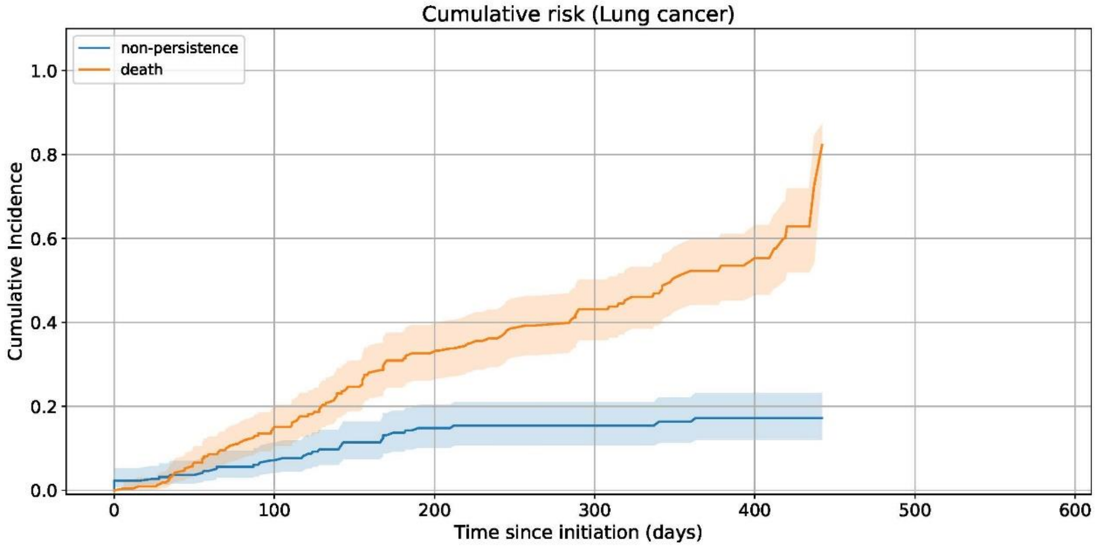

Event Counts							
At Risk	212.0	151.0	89.0	63.0	15.0	0.0	0.0
Censored	4.0	24.0	40.0	48.0	81.0	91.0	91.0
Non-persistence	6.0	16.0	30.0	31.0	33.0	33.0	33.0
Death	0.0	31.0	63.0	80.0	93.0	98.0	98.0

d

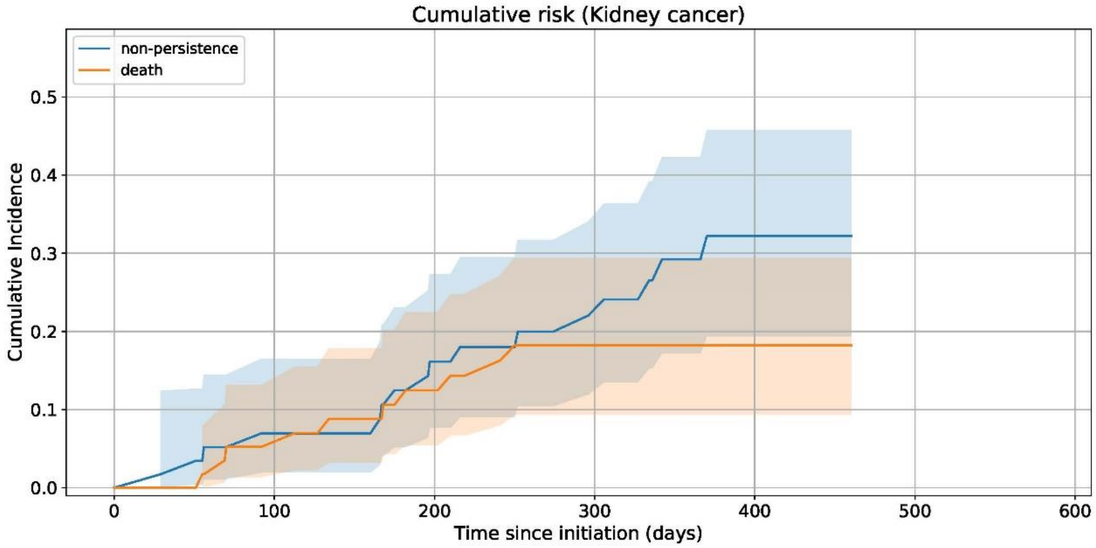

Event Counts							
At Risk	57.0	49.0	38.0	28.0	8.0	0.0	0.0
Censored	1.0	2.0	4.0	8.0	24.0	32.0	32.0
Non-persistence	1.0	5.0	10.0	13.0	17.0	17.0	17.0
Death	0.0	3.0	7.0	10.0	10.0	10.0	10.0

e

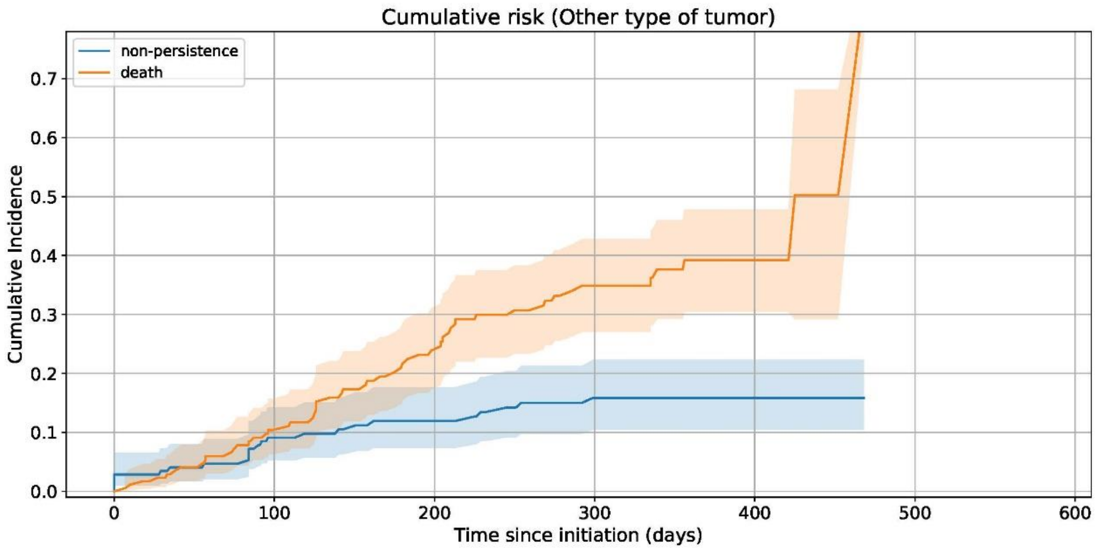

Event Counts

At Risk	167.0	122.0	85.0	56.0	9.0	0.0	0.0
Censored	5.0	23.0	37.0	47.0	91.0	98.0	98.0
Non-persistence	6.0	16.0	20.0	25.0	25.0	25.0	25.0
Death	0.0	17.0	36.0	50.0	53.0	55.0	55.0

Supplementary Fig. 3 Cumulative risk of non-persistence. Numbers at risk are shown in **Supplementary Fig. 2**. Solid lines represent cumulative incidence

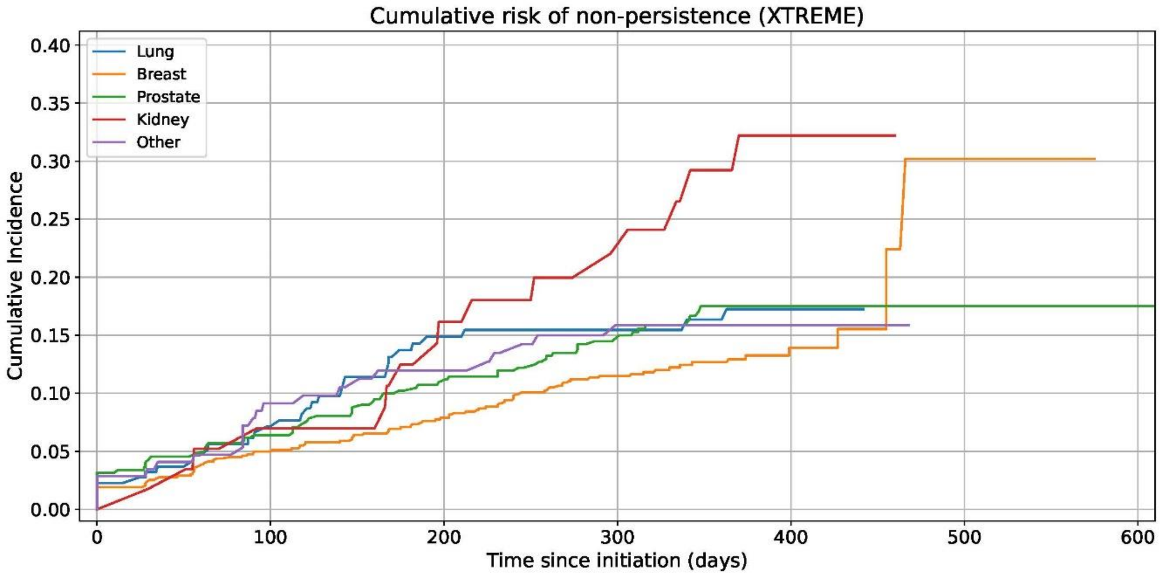

Supplementary Fig. 4 Cumulative risk of non-persistence and death (breast cancer). Solid lines represent cumulative incidence; surrounding shaded areas represent 95% confidence intervals

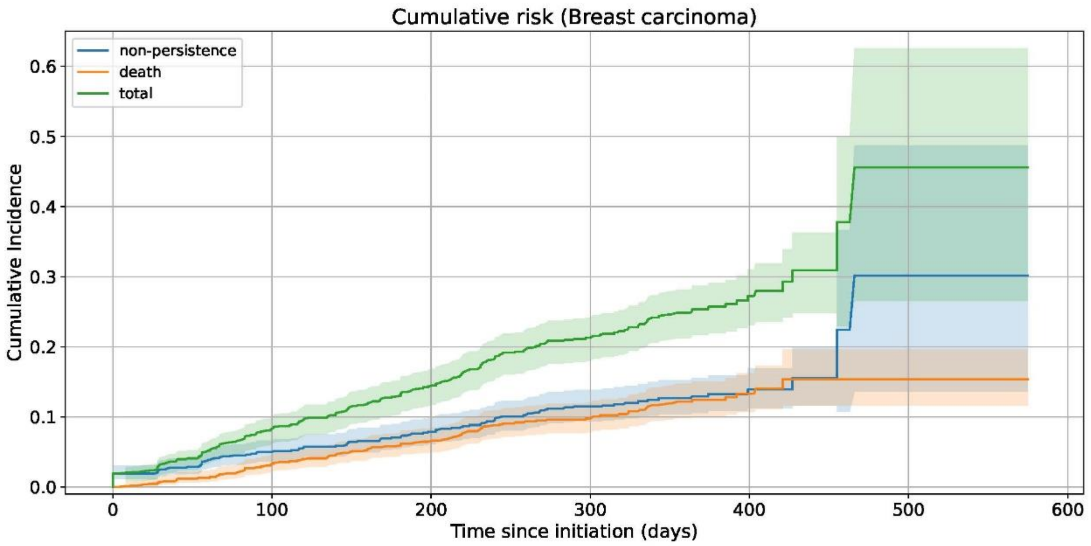

Event Counts

At Risk	816.0	721.0	631.0	537.0	103.0	3.0	0.0
Censored	10.0	53.0	97.0	141.0	551.0	646.0	649.0
Non-persistence	17.0	42.0	64.0	90.0	99.0	102.0	102.0
Death	0.0	27.0	51.0	75.0	90.0	92.0	92.0

References

1. De Geest S, Zullig LL, Dunbar-Jacob J, Helmy R, Hughes DA, Wilson IB, Vrijens B (2018) ESPACOMP Medication Adherence Reporting Guideline (EMERGE). Ann Intern Med 169:30-35. https://doi.org/10.7326/m18-0543

2. Coleman R, Hadji P, Body JJ, Santini D, Chow E, Terpos E, Oudard S, Bruland Ø, Flamen P, Kurth A, Van Poznak C, Aapro M, Jordan K (2020) Bone health in cancer: ESMO Clinical Practice Guidelines. Ann Oncol 31:1650-1663. https://doi.org/10.1093/annonc/mdu103

3. Diel I, Janssen J, Kluike C, Schilling J (2018). Observational study on the usage of denosumab for prevention of skeletal related events (SREs) in patients with bone metastases and solid tumors in routine clinical practice (X-TREME). Presented at Annual Meeting of the German, Austrian and Swiss Societies of Hematology and Medical Oncology; Vienna, Austria: Poster 69

4. Haslbauer F, Petzer A, Safanda M, Tomova A, Porubska M, Bajory Z, Niepel D, Jaeger C, Bjorklof K, Kalinin D, Greil R (2020) Prospective observational study to evaluate the persistence of treatment with denosumab in patients with bone metastases from solid tumors in routine clinical practice: final analysis. Support Care Cancer 28:1855-1865. https://doi.org/10.1007/s00520-019-04988-7
